# Magnetic Field Dependence of AC Susceptibility of Ho_2−*x*_*M_x_*Ti_2_O_7_ (*M* = Bi, Y) Titanates

**DOI:** 10.3390/ma19214655

**Published:** 2026-10-31

**Authors:** Anatoly B. Rinkevich, Dmitry V. Perov, Maria S. Koroleva, Irina V. Piir

**Affiliations:** 1 M.N. Miheev Institute of Metal Physics UB RAS, Sofia Kovalevskaya St., 18, 620108 Ekaterinburg, Russia; 2 Institute of Chemistry UB RAS, Pervomayskaya St., 48, 167000 Syktyvkar, Russia

**Keywords:** rare earth titanate pyrochlores, AC magnetic susceptibility, differential DC magnetic susceptibility, modified Cole–Cole formula, magnetic relaxation time

## Abstract

The dependence of magnetic susceptibility in alternating fields (AC susceptibility) of rare earth titanates Ho_2−*x*_*M_x_*Ti_2_O_7_ (*M*—Bi, Y) on the magnetic field was studied. The measurements were performed in the frequency range from 1 Hz to 1 kHz at temperatures from 1.8 K to 20 K. The conformity of the field dependence of AC susceptibility to the modified Cole–Cole formula was verified. It has been established that the characteristic field in which the real part of the AC susceptibility decreases by a factor of 2 rapidly increases with increasing temperature. A correlation was established between the field dependence of the real part of the AC susceptibility and the differential susceptibility. From the frequency dependence of the AC susceptibility, the temperature dependence of the magnetic relaxation time was determined.

## 1. Introduction

The unusual magnetic properties and exchange interactions in rare earth titanates have been the subject of research in recent years. The problem of the realization of the spin ice state, in which numerous degrees of freedom under the action of frustrated interactions prevent the establishment of magnetic ordering, is being carefully studied [1]. In the spin ice state, there exist deconfined “magnetic monopoles” capable of demonstrating the quantum spin dynamics [2,3]. The spin ice state was observed in dysprosium Dy_2_Ti_2_O_7_ and holmium Ho_2_Ti_2_O_7_ titanates. Rare earth ions are located at the sites of tetrahedrons in the crystal lattice of pyrochlores. In the spin ice state, the stable arrangement of magnetic moments is inside the tetrahedron for two ions and outside the tetrahedron for the other two. The study of AC magnetic susceptibility is one of the effective ways to investigate the spin dynamics of rare earth titanates [4]. Experiments on AC susceptibility and magnetic relaxation in Dy_2_Ti_2_O_7_ at low temperatures showed that the spin dynamics are described by two relaxation times [5]. The magnetic relaxation time increases with decreasing temperature [6]. The existence of two relaxation times in titanate Ho_2_Ti_2_O_7_ was confirmed in [7]. At the lowest temperatures, the relaxation mechanism associated with the motion of monopoles predominates, while at higher temperatures, relaxation occurs due to the formation of bound states of monopoles. In a study of highly pure Ho_2_Ti_2_O_7_ crystals in [7], it was established that there is a thermal crossover between these two types of relaxation dynamics. Two magnetic relaxation times were observed from the frequency dependence of the AC susceptibility of Yb_2_Ti_2_O_7_ nanocomposite [8]. The two-time magnetic relaxation model was found to be necessary to describe the frequency dependence of nanocomposite titanates Gd_2_Ti_2_O_7_, Yb_2_Ti_2_O_7_, Dy_2_Ti_2_O_7_, and Dy_2_Si_2_O_7_ [9]. Spin dynamics in Y and Bi-doped rare earth holmium titanates are studied in detail in [10]. To describe the magnetic relaxation of these compounds, a model of two magnetic relaxation times is also needed.

Doping of rare earth titanate with ions of other elements with the same valence, but having an ionic radius different from that of the rare earth ion, is often considered to lead to a “chemical pressure” effect. The doping ion replaces the rare earth ion in the crystal lattice and, due to the difference in the ion radius, changes the distance between neighboring ions and changes the lattice parameter. Doping of Yb_2_Ti_2_O_7_ with Sn, Ti, and Ge ions was investigated in [11]. It is shown that doping can lead to the establishment of either ferromagnetic or antiferromagnetic ordering. Doping of holmium titanate with germanium leads to the fact that germanium ions occupy the positions of titanium in the crystal lattice, which leads to a change in the structure of the compound and to a change in the electronic structure and optical properties [12]. Substitution of the nonmagnetic Ti ion in the crystal lattice with Fe^3+^ ions leads to modification of optical properties [13]. The doping of dysprosium titanate with nonmagnetic calcium was studied in [14] by examining the temperature dependences of AC and DC susceptibilities. The analysis of Cole–Cole plots showed that in calcium-doped dysprosium titanates, there is a single magnetic relaxation time. The effect of doping of ytterbium and holmium titanates with yttrium and bismuth on magnetic properties was studied in [15,16]. In [16], the magnetization curve and the temperature dependence of the DC susceptibility are studied in detail. Data on the g-factor are obtained from the magnetization curves. The magnetic dipole and exchange interactions of titanates are analyzed from the temperature dependences of magnetic susceptibility. A review of data on temperature dependences of magnetic susceptibility, crystal field, and exchange interactions in rare earth titanates with a pyrochlore structure is contained in [17]. The effect of europium doping of dysprosium titanate performed in [18] showed that all phonon modes shift with increasing europium content. The study of AC susceptibility has shown that in samples with a high content of europium, there are two transitions at which spin freezing occurs.

The dependence of AC susceptibility on the magnetic field strength has been less studied to date. Ref. [2] shows how, using measurements of the frequency dependences of AC susceptibility to obtain data on the relaxation time and mobility of magnetic monopoles. The application of a magnetic field made it possible to establish a difference in the ground state of ytterbium titanate doped with tin, titanium, and germanium [11]. The study of the AC susceptibility of paramagnet Tb_2_Ti_2_O_7_ in a strong magnetic field showed the presence of very slow spin relaxation, so that even in the paramagnetic state, the cooperative behavior of spins is carried out [19]. The effect of the magnetic field on the spin freezing transition temperature was studied in [6] for Dy_2_Ti_2_O_7_. The spin dynamics is described by the relaxation time distribution, which narrows at the lowest temperatures. To describe the dependence of AC susceptibility on the magnetic field strength in rare earth titanate pyrochlores, a formula similar to the Cole–Cole formula for the frequency dependence of susceptibility was proposed [20,21]. This formula successfully describes the field dependence of AC susceptibility for the nanocomposite titanate Yb_2_Ti_2_O_7_ [8].

In this article, the dependences of AC susceptibility on the magnetic field and frequency will be investigated. The study was conducted with holmium titanate doped with bismuth and yttrium. In doped compounds, the radius of Bi^3+^ (1.17 Å) ion differs significantly from the radius of Ho^3+^ (1.015 Å), whereas the radius of the Y^3+^ ion (1.019 Å) is close to it [22]. Therefore, it can be assumed that bismuth doping creates chemical pressure, while yttrium doping does not. Various, predominantly high, levels of yttrium doping were selected—specifically, *x* = 1.5, 1.75, and 1.9. These doping levels were chosen with the aim of exerting a greater influence on the distribution of Ho^3+^ ions. A single concentration was sufficient for comparing the effects of dopants with different ionic radii. For the purpose of comparing the effects of yttrium and bismuth doping, a value of *x* = 1.5 was selected. Both doping ions have no magnetic moment. The article studies the field dependences of AC susceptibility at different temperatures for samples with different degrees of doping. The study of the nature of the field dependence of the AC susceptibility constitutes the main objective of this paper. The article will investigate the temperature dependence of the characteristic field associated with the real part of the AC susceptibility for undoped Ho_2_Ti_2_O_7_ and doped Ho_2−*x*_*M_x_*Ti_2_O_7_ (*M* = Bi, Y) compositions. The AC susceptibility will be compared with the differential DC susceptibility, which is calculated by measuring the magnetization curves. For Ho_2_Ti_2_O_7_ and yttrium-doped compositions, the existence of a correlation between *dM*/*dH* and χ′(*H*) will be examined. The frequency dependences of AC susceptibility have been studied and the temperature dependence of the magnetic relaxation times has been obtained.

## 2. Samples and Methods

**Figure 1:**
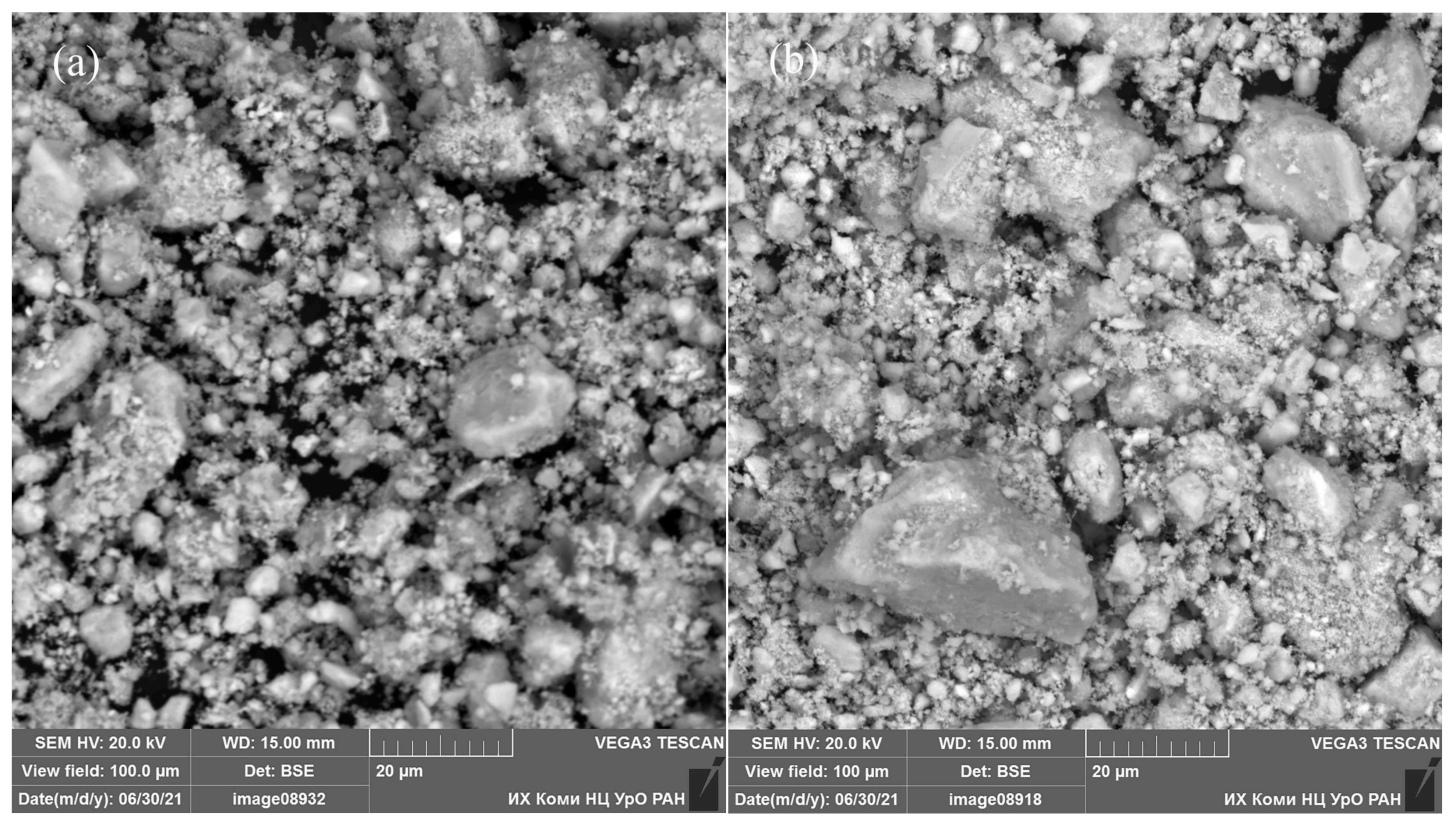
Micrograph of Y_1.75_Ho_0.25_Ti_2_O_7_ (**a**) and Y_1.5_Ho_0.5_Ti_2_O_7_ (**b**) powder obtained using Tescan Vega 3SBU type scanning microscope.

Compositions of Ho_2−*x*_*M_x_*Ti_2_O_7_ (*M*—Bi, Y) were obtained. The solid-state reaction method, described in detail in [16], was used for the synthesis of Ho_2−*x*_Y*_x_*Ti_2_O_7_ (*x* = 1.5; 1.75) and Bi_1.5_Ho_0.5_Ti_2_O_7_. Their final calcination was carried out at 1500 °C (3 h) for Ho_2−*x*_Y*_x_*Ti_2_O_7_ and 1150 °C (20 h) for Bi_1.5_Ho_0.5_Ti_2_O_7_. The coprecipitation method was used to synthesize Ho_2−*x*_Y*_x_*Ti_2_O_7_ (*x* = 0; 1.9), with the final calcination performed at 1000 °C for 10 h. The coprecipitation procedure corresponded to that described in [23]. The samples obtained via the solid-state method are characterized by larger agglomerates compared to samples obtained via the coprecipitation method, which is due to the high temperature used for their final calcination. The X-ray diffraction and scanning electron microscopy data showed that all samples were almost single-phase. Even with a high degree of doping with Y or Bi, they retain the pyrochlore structure. The composition of the compounds, as determined by energy-dispersive X-ray microanalysis (EDS), corresponds to the initially specified composition within the margin of error of the method (2–3%). The synthesis of the compounds was carried out in an air atmosphere, in which the Ho^3+^, Y^3+^ and Bi^3+^ cations are thermodynamically stable; this also prevents the reduction of titanium from the Ti^4+^ to the Ti^3+^ state. Thus, the stability of the oxidation states of the cations and the strict adherence to their stoichiometric ratio make it possible to conclude that the oxygen content in the resulting compounds corresponds to the stoichiometric value. Figure 1 shows the results of scanning electron microscopy of Y_1.75_Ho_0.25_Ti_2_O_7_ and Y_1.5_Ho_0.5_Ti_2_O_7_ powders. Microscopy was performed on a Tescan Vega 3SBU type microscope (Tescan Orsay Holding, Brno, Czech Republic) at an accelerating voltage of 20 kV. The powders contain particles ranging in size from 0.2 to 20 µm. The particle shape is irregular, and the orientation is random. The powders were pressed into tablets of approximately 15 mm in diameter. For the Ho_0.5_Y_1.5_Ti_2_O_7_ composition, for example, porosity was determined to be 4–5%. The presence of impurities does not exceed 2%. The impurities consist of titanates of a different composition and do not contain other ions with a magnetic moment. In the pyrochlore structure with the composition Y_2−*x*_Ho_*x*_Ti_2_O_7_ or Bi_2−*x*_Ho_*x*_Ti_2_O_7_, the holmium dopant occupies the yttrium or bismuth sites. According to the literature, even in dilute solid solutions with a pyrochlore structure, holmium (at *x* > 0.1) tends to form clusters containing both transition d-elements and rare earth elements [24,25,26].

**Figure 2:**
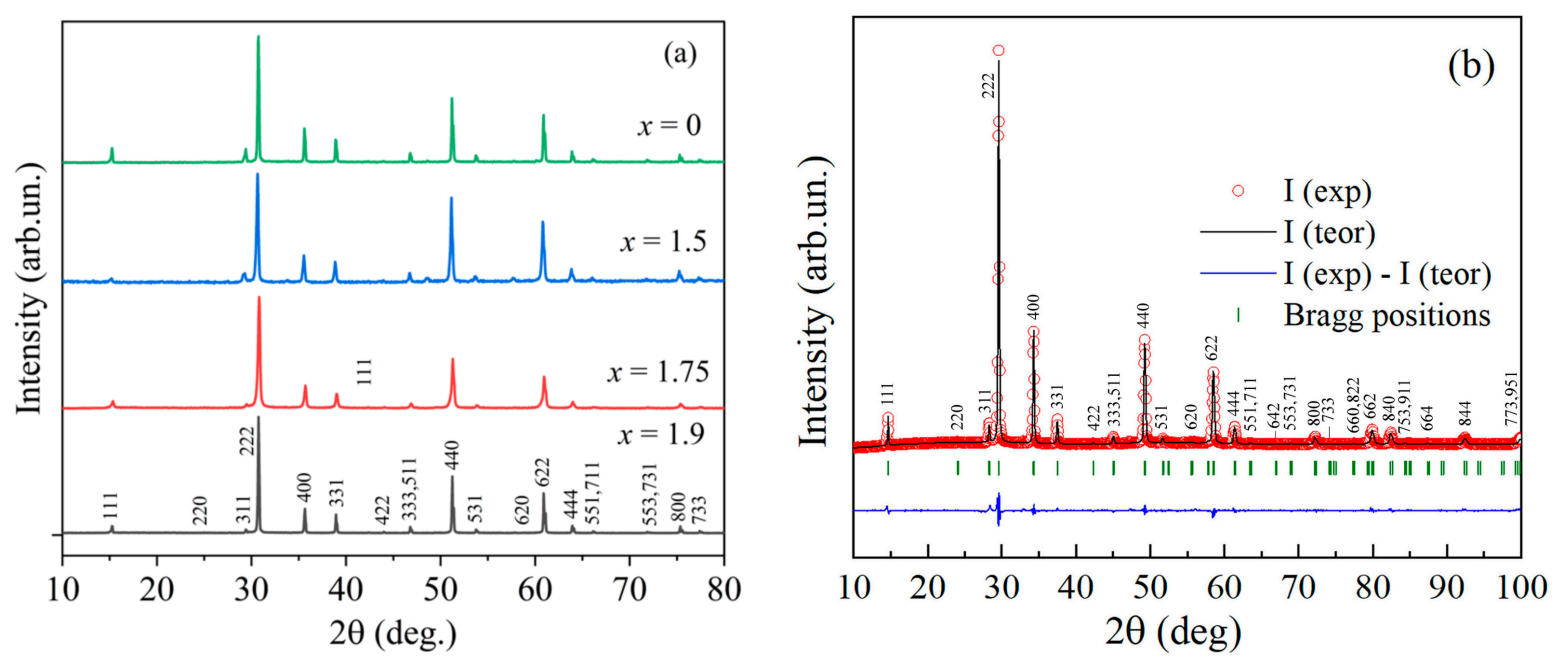
The X-ray diffraction patterns for Ho_2−*x*_Y*_x_*Ti_2_O_7_ powders (**a**) and the pattern for Bi_1.5_Ho_0.5_Ti_2_O_7_ (**b**). Rietveld results are shown in (**b**) for the powder XRD pattern.

The X-ray diffraction data are shown in Figure 2. The analysis was performed on a Shimadzu XRD-6000 diffractometer (Shimadzu Corp., Cioto, Japan) with an emission wavelength of 1.54056 Å, a 2*θ* scanning range from 10 to 100° and a step of 0.05°. The crystal lattice parameter a for Ho_2_Ti_2_O_7_ was determined in several studies. In [27] the value a as 10.102 Å was obtained, and according to [28] a = 10.09832(1) Å. In the article [29], a fairly close value of a = 10.103421(1) Å was obtained. The values of the lattice parameter a, the distances between the rare earth ion R and another R ion, or the doping ion Bi or Y, and the X-ray density are given in Table 1. These data are quite close to those published in [22,30]. The calculation of the cell parameters and X-ray density of the samples was carried out using the FullProf software package (Classic, Institut Laue-Langevin, Grenoble, France). When refining the crystal structure of holmium-containing pyrochlores using the Rietveld method, the agreement factors between the experimental and theoretical X-ray diffraction patterns are found to be higher than those for analogous compounds containing lighter elements. This is due to strong absorption and microabsorption by holmium, which leads to a reduction in the intensity of diffraction peaks at high 2θ angles and a distortion of the reflection profiles resulting from microabsorption effects. The fluorescence effect leads to a significant increase in the background level and a reduction in the signal-to-noise ratio. The experimental X-ray diffraction reflections were described using the Thompson–Cox–Hastings pseudo-Voigt model (Extended pseudo-Voigt), incorporating an axial divergence asymmetry component, with the peak asymmetry parameter being refined. The results of the Rietveld refinement for the composition of bismuth-doped holmium titanate are presented in Figure 2b.

**Figure 3:**
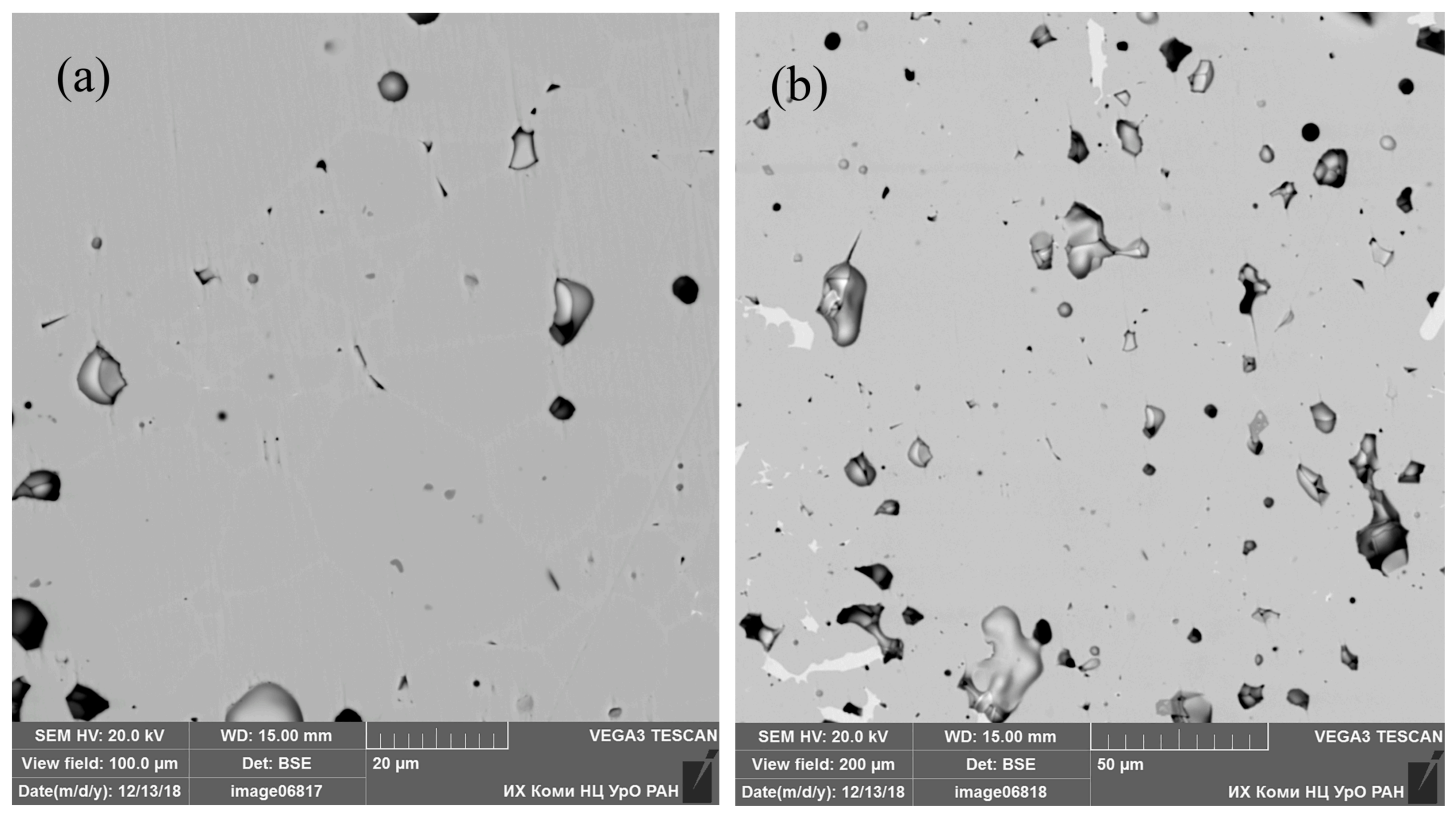
The BSE SEM images ((**a**) and (**b**)) for Bi_1.5_Ho_0.5_Ti_2_O_7_ (EDX: main phase is Bi_1.52_Ho_0.48_Ti_2_O_7_, minor phase (light in micrograph) is Bi_4_Ti_2.75_Ho_0.27_O_12-δ_).

The porosity of the samples and the proportion of impurities were determined from SEM micrographs taken at various locations (Figure 3). The volume fraction of the primary phase and the impurity phase, as well as the porosity, were determined based on the relative areas of regions with different contrast levels corresponding to the phases present, as observed in BSE mode.

The content of impurities can also be determined from X-ray diffraction patterns, provided that the primary reflections of the impurity phase do not overlap with those of the main phase. For the compounds obtained, no reflections corresponding to impurity phases are observed in the X-ray diffraction data (the accuracy of the X-ray diffraction analysis is about 2%). Based on the SEM micrographs presented in Figure 1 for the compositions obtained via the coprecipitation method, the phase contrast is uniform, which indicates that the compounds are single-phase. In the composition containing bismuth, an additional impurity phase—a layered perovskite—is present; it is clearly visible in the micrograph as bright regions.

## 3. DC Magnetic Measurements

**Figure 4:**
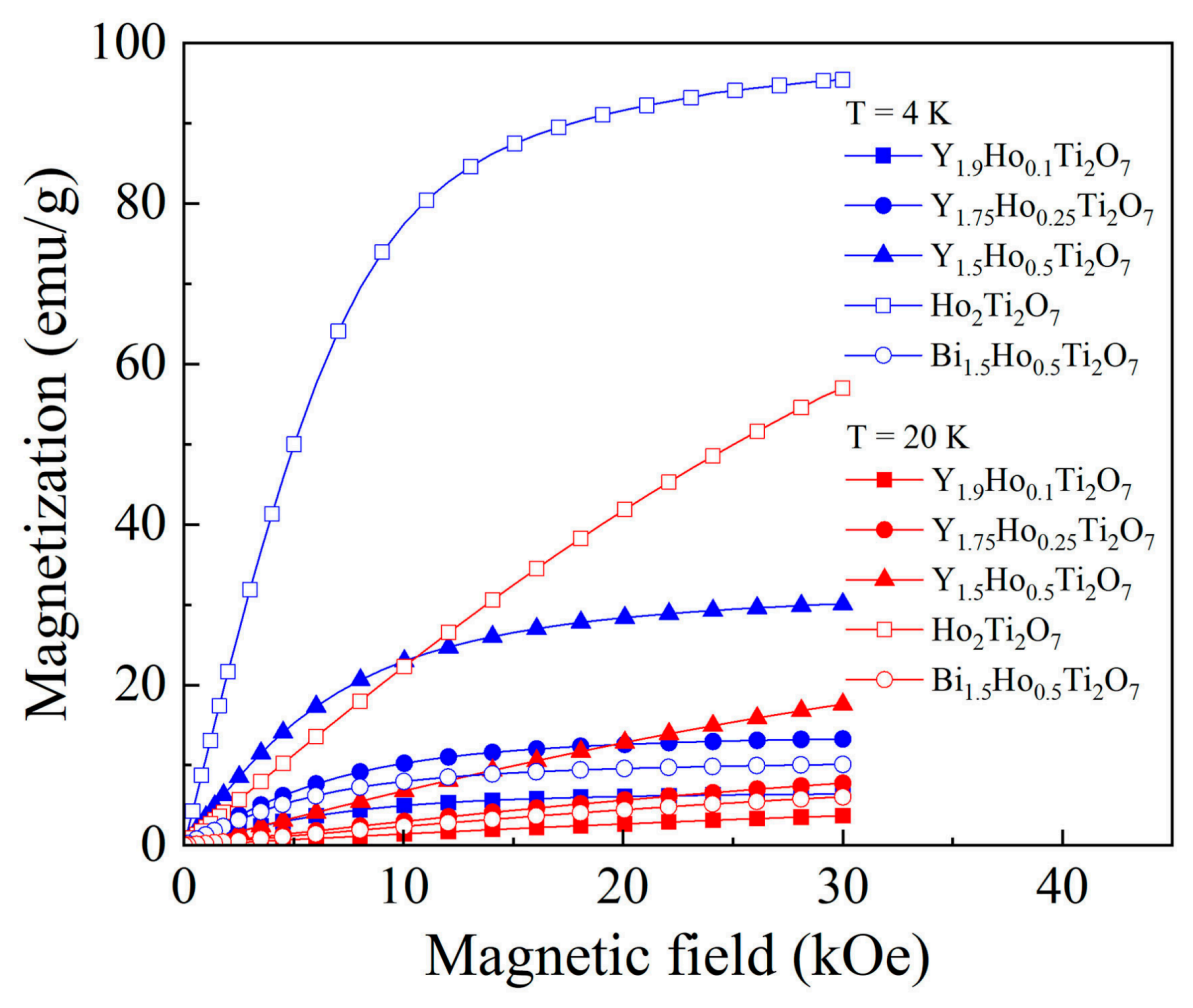
Magnetization curves of doped holmium titanates measured at temperatures of 4 K and 20 K.

DC magnetic measurements were performed on a MPMS-XL-5 SQUID magnetometer (Quantum Design, Inc., San Diego, CA, USA) at temperatures *T* from 2 to 20 K. Magnetization curves were measured in fields up to 30 kOe. The magnetization measurement error does not exceed 5%. Figure 4 shows the results of measurements at temperatures of 4 K and 20 K. The dependences measured at *T* = 4 K show signs of saturation, while the dependences measured at *T* = 20 K demonstrate a dependence close to linear. Of course, doping titanate with an element that does not have a magnetic moment leads to a decrease in magnetization.

Magnetization curves are needed in this article to construct field dependences of differential susceptibility. This is addressed below in the Discussion Section and they will be compared with the field dependencies of AC susceptibility. The magnetization curves of doped holmium titanates were studied in detail in the article [16]. To do this, it is necessary to recalculate the data shown in Figure 3 so that the magnetic moment per Ho^3+^ ion is plotted along the ordinate axis. The calculation must take into account the number of ions per 1 cm^3^ of the substance at a given doping level, the density of the material, and the porosity of the sample. This was conducted in [16]. It was shown there that doping with both bismuth and yttrium leads to practically identical changes in the magnetic moment per 1 ion of the rare earth element. So, the chemical pressure during bismuth doping did not have a significant effect. The magnitude of the saturation magnetization in undoped and doped holmium titanates corresponds to the direction of the magnetic moments of rare earth ions, which is different from that realized for spin ice. Here, within one tetrahedron of the crystal structure, three moments are directed out of the tetrahedron and one inward. For the neighboring tetrahedron, on the contrary, three moments are directed inward and one outward. This arrangement of magnetic moments in a moderately strong magnetic field (H~10–30 kOe) was established in [31] for Ho_2_Ti_2_O_7_ and in [16] for doped holmium titanates.

## 4. AC Magnetic Measurements

**Figure 5:**
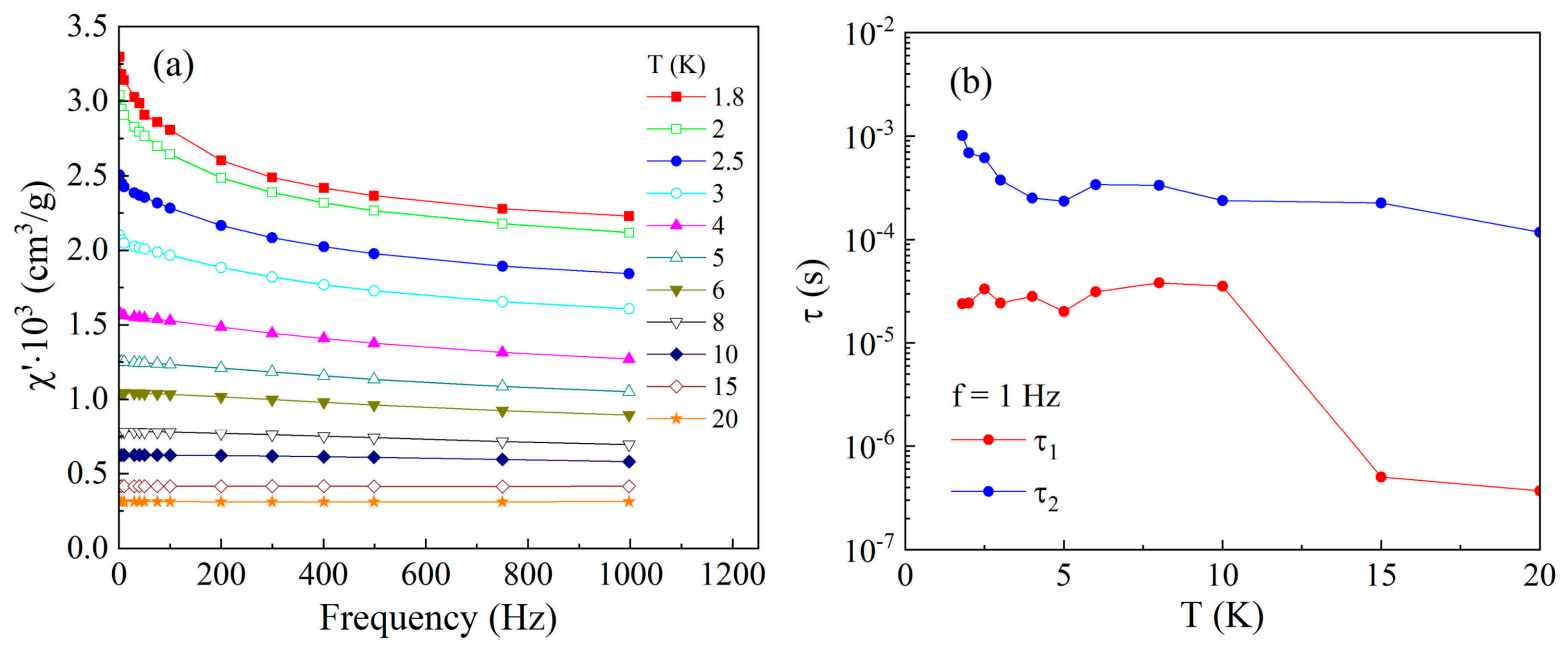
Frequency dependence of the real part of the magnetic susceptibility measured at several temperatures (**a**) and temperature dependence of the relaxation times (**b**) for the Ho_0.25_Y_1.75_Ti_2_O_7_ sample.

AC magnetic measurements were performed on an MPMS-XL-5 SQUID magnetometer at temperatures from 1.8 to 20 K in DC magnetic fields up to 50 kOe. Frequencies *f* from 1 Hz to 1 kHz were used. The amplitude of the alternating field during measurements was $H_{\sim }$ = 4 Oe. The frequency dependence of the real part of the magnetic susceptibility $\chi^{\prime }$, measured at H = 0 for a sample of composition Ho_0.25_Y_1.75_Ti_2_O_7_, is shown in Figure 5a. With increasing frequency, $\chi^{\prime }$ decreases and tends to some constant value corresponding to the adiabatic limit $\chi \left(f\to \infty \right)$. With increasing temperature, the magnitude of changes in the frequency dependence $\chi^{\prime }\left(f\right)$ becomes smaller.

**Figure 6:**
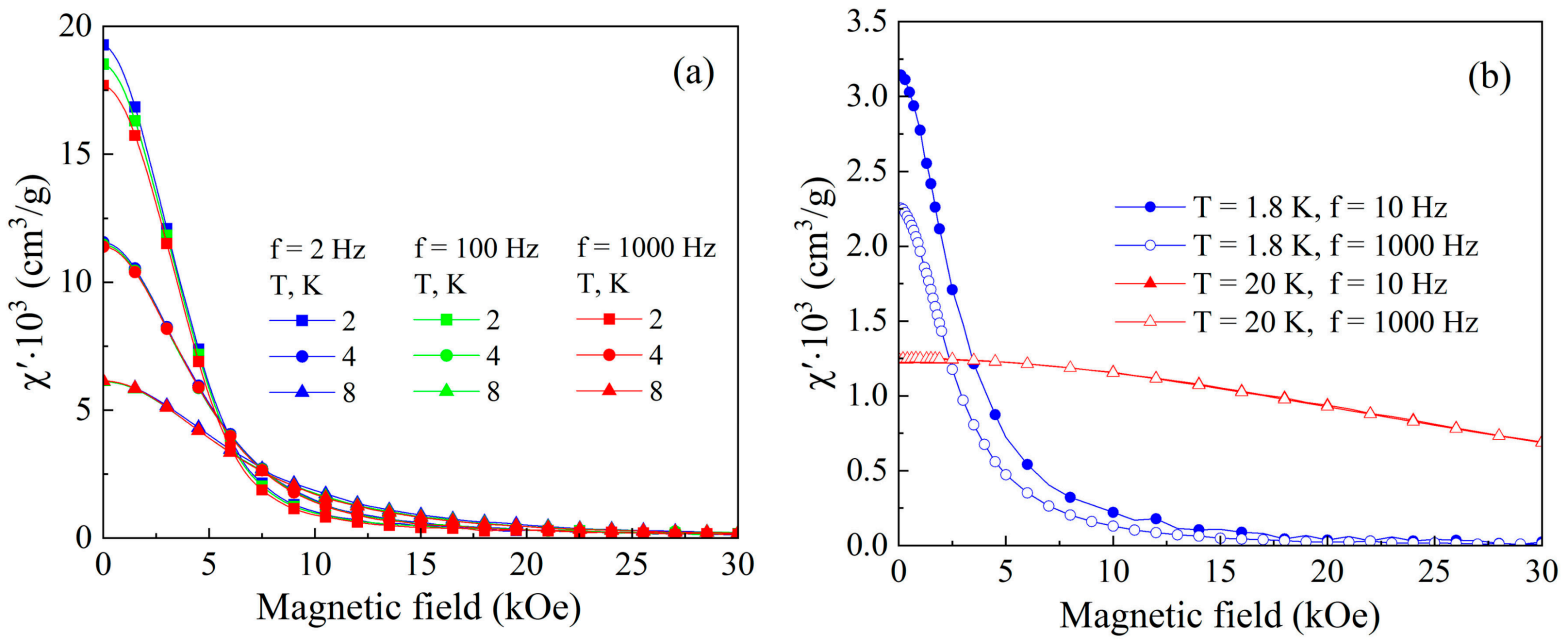
The dependences of the real part of the AC magnetic susceptibility of titanates on the magnetic field at different frequencies and different temperatures: Ho_2_Ti_2_O_7_—(**a**); Ho_0.25_Y_1.75_Ti_2_O_7_—(**b**).

Figure 6 shows the dependences of the real part of the AC susceptibility for Ho_2_Ti_2_O_7_ and the doped titanate Ho_0.25_Y_1.75_Ti_2_O_7_. The dependences in Figure 6a were measured at three temperatures 2 K, 4 K, 8 K and three frequencies 2 Hz, 100 Hz and 1000 Hz. With increasing magnetic field strength, the real part of the AC susceptibility decreases significantly. The influence of the alternating field frequency is better seen in Figure 6b. At a low temperature of *T* = 1.8 K it is evident that increasing the frequency from 10 Hz to 1000 Hz leads to a decrease in $\chi^{\prime }$. At a high temperature of *T* = 20 K the influence of frequency is almost imperceptible.

## 5. Discussion

To analyze the frequency dependence of the real part of the AC susceptibility shown in Figure 5a, we use the Cole–Cole model [21]. The response of the system to an external alternating field is modeled by a system of *K* oscillators, the resonant frequencies of which $f_{k}$ are inversely proportional to the relaxation times of the spins $\tau_{k}$, $f_{k}=1/\left(2\pi \tau_{k}\right)$. The Cole–Cole formula, which describes the frequency dependence of complex susceptibility, is as follows:

(1) $$\dot{\chi }\left(\omega \right)=\sum_{k=1}^{K}\left(\chi_{k}^{\infty }+\frac{\chi_{k}^{0}-\chi_{k}^{\infty }}{1+{\left(i\omega \tau_{k}\right)}^{1-\alpha_{k}}}\right)$$

where ω=2πf is the circular frequency, $\;\chi_{k}^{0}$ is isothermal magnetic susceptibility at ω→0, and $\;\chi_{k}^{\infty }$ is adiabatic magnetic susceptibility in the high-frequency limit. The relative width $\alpha_{k}$ of the frequency distribution, corresponding to the oscillator *k*, is a positive value less than 1, where $0\leq \alpha_{k}<1$. The approximation data for the dependencies shown in Figure 5a are given in Table 2.

As shown in [10], for the approximation it is necessary to use two spin relaxation times $\tau_{1}$ and $\tau_{2}$. The data in this article differ from [10] by a wider temperature range. In the paper [32], the presence of only a single relaxation time was established. Nevertheless, the text of the article [32] mentions that two processes coexist with similar time scales but different thermal evolution. The existence of two spin relaxation times has been quite reliably established in the article [7]. The values of the corresponding parameters for the first and second oscillators are shown in Table 2 using a double slash. From Table 2 it can be seen that the isothermal susceptibility for the first oscillator is greater than for the second, with $\chi_{1}^{0}>\chi_{2}^{0}$ for all temperatures. The adiabatic susceptibility for the second oscillator is greater than for the first, with $\chi_{2}^{\infty }>\chi_{1}^{\infty }$ at temperatures T < 6 K. At higher temperatures, the opposite inequality is observed. The results of determining the relaxation time for two oscillators are shown in the graph in Figure 5b. It can be seen that the time τ_1_ changes noticeably in the temperature range of 10–20 K, and changes weakly at lower temperatures. A slight change in the spin relaxation time in the temperature range of 2–10 K was observed for dysprosium titanate [6]. The temperature dependence of the relaxation time τ_1_ is consistent with the predictions of the Debye–Hückel theory within a monopole excitation model [33]. In the temperature range 2–10 K, a weak temperature dependence was observed, as predicted by the Debye–Hückel theory for this range. At temperatures above 10 K, the relaxation time values decrease sharply; in [33], this is attributed to a departure from the assumption—central to the Debye–Hückel theory—that the system can be described as a gas of weakly interacting particles. At temperatures above 10 K, a single-ion thermally activated relaxation process takes place. The time $\tau_{2}$ corresponds to a slower relaxation of spins. At temperatures T < 6 K, the parameter $\alpha_{2}$ has a noticeable value when $\alpha_{2}$ > 0.1. The parameter α_1_ for fast relaxation in most cases turned out to be small, where $\alpha_{1}$ < 10^−3^ except for temperatures 2.5 K, 6 K and 8 K. The values of $\alpha_{1}$ and $\alpha_{2}$ that are less than 10^−3^ are obtained as a result of the approximation algorithm, but they are not reliable, actual values. In [7], when analyzing the AC susceptibility data over a wide range of frequencies and temperatures, it was found that at temperatures below the crossover temperature (*T* = 1.2 K for Ho_2_Ti_2_O_7_), relaxation occurs as a result of quantum tunneling of thermally activated monopoles at their low density. This relaxation mechanism corresponds to relaxation times that rapidly increase with decreasing temperature. At higher temperatures, according to [7,33,34], coordinated motion of pairs of monopoles occurs. Monopoles and antimonopoles contain spin direction configurations of the tetrahedron “three out–one in” for one and “three in–one out” for the other [31]. The motion of pairs of monopoles corresponds to a faster relaxation [7]. This type of relaxation can persist up to temperatures of a few Kelvins. In the doped titanate Ho_0.25_Y_1.75_Ti_2_O_7_, one can only tentatively attribute relaxation to the motion of monopoles, even at low temperatures. Since the concentration of Ho^3+^ ions is below the percolation threshold, the motion of monopoles can only occur within regions of non-uniform ion distribution—that is, within clusters. At temperatures above 10 K, the relaxation time τ_1_ in this titanate decreases sharply, which indicates a change in the relaxation mechanism. As described in [35], a single-ion thermally activated relaxation process takes place.

**Figure 7:**
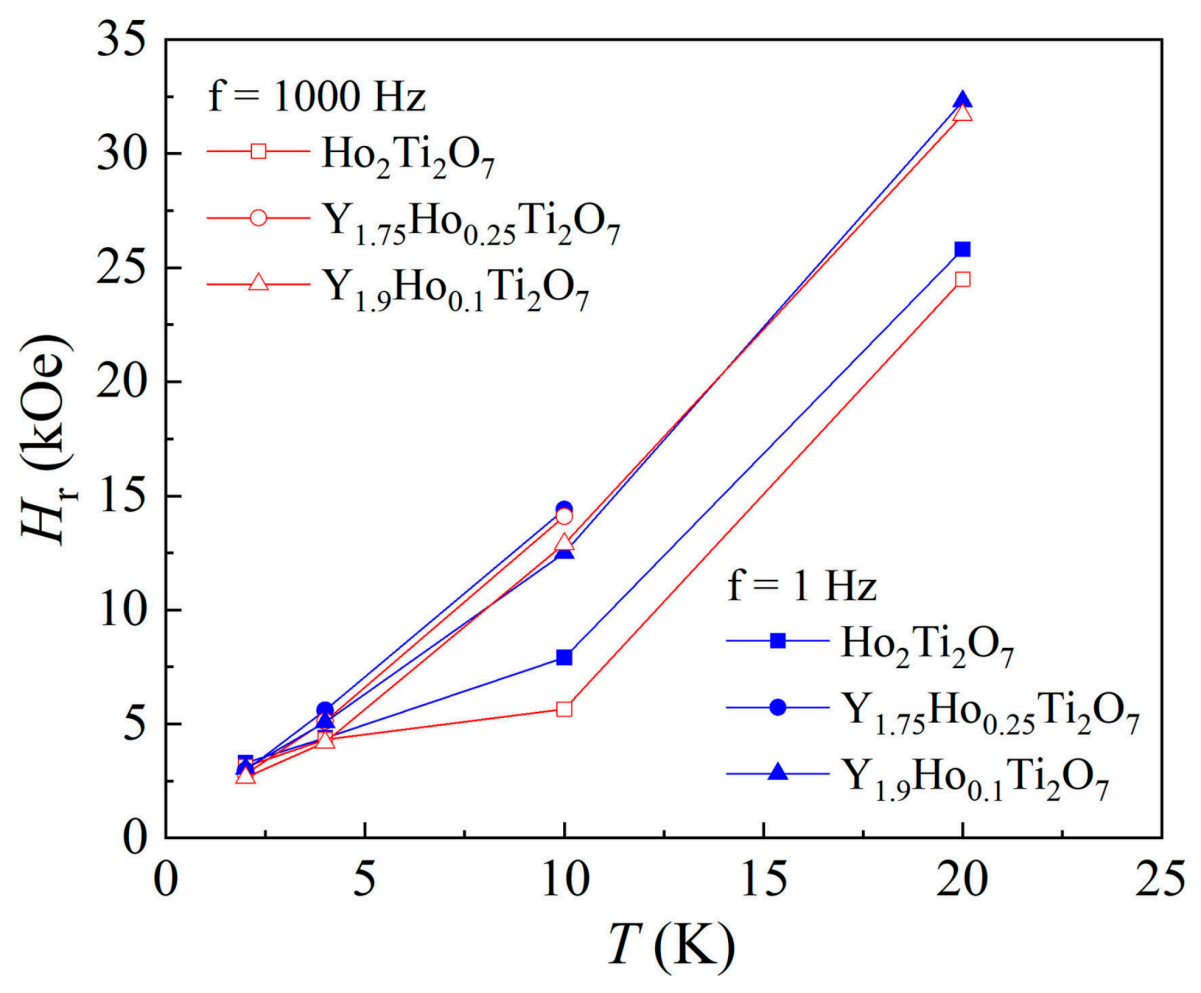
Temperature dependence of the *H_r_* field of doped holmium titanates.

We will now move on to a discussion of the field dependence of AC susceptibility. From Figure 6 it can be seen that as DC magnetic field strength increases, $\chi^{\prime }$ decreases. Let us introduce the field $H_{r}$, for which $\chi^{\prime }\left(H_{r}\right)=\frac{1}{2}\left(\chi^{\prime }\left(0\right)-\chi^{\prime }\left(H_{\max}\right)\right)$, where $H_{\max}$ is the maximum field used in the measurements. In all cases in our measurements, it turned out that $\chi^{\prime }\left(0\right)>>\chi^{\prime }\left(H_{\max}\right)$. It was found that the $H_{r}$ field is strongly dependent on temperature. The results for several titanates are shown in Figure 7. With increasing temperature, the magnitude of the $H_{r}$ field increases. This trend is present for both undoped holmium titanate and doped titanates. The measurement frequency has only a minor effect on the temperature dependence of the $H_{r}$.

In [20], a formula was proposed to describe the field dependence of the real part of the AC susceptibility of rare earth titanates. In its written form it is similar to the Cole–Cole formula (1), but the quantities included in it have a different meaning. The modified formula for Cole–Cole is given below:

(2) $$\chi \left(H\right)=\chi^{\prime }\left(H\right)-i\chi^{\prime \prime }\left(H\right)=\chi_{\infty }+\frac{\chi_{0}-\chi_{\infty }}{1+{\left(i\frac{H}{H_{r}}\right)}^{1-\alpha }}$$

where $\chi_{0}$ is the dynamic susceptibility at H=0 and $\chi_{\infty }$ is the dynamic susceptibility at H→∞. The field $H_{r}$ enters into (2). Its physical meaning is easily seen when α=0. When $H=H_{r}$, the real part of the susceptibility decreases by a factor of two compared to the value at H=0. From (2) we can obtain an expression for the real part of $\chi^{\prime }$:

(3) $$\chi^{\prime }=\frac{\chi_{0}\left(1+{\left|\frac{H}{H_{r}}\right|}^{1-a}\cos\left(\frac{\pi }{2}\left(1-a\right)\right)\right)}{D_{x}}$$

where

$$D_{x}={\left(1+{\left|\frac{H}{H_{r}}\right|}^{1-a}\cos\left(\frac{\pi }{2}(1-a)\right)\right)}^{2}+{\left({\left|\frac{H}{H_{r}}\right|}^{1-a}\sin\left(\frac{\pi }{2}\left(1-a\right)\mathrm{sgn}\left(\frac{H}{H_{r}}\right)\right)\right)}^{2}$$

When α=0 and $\chi_{0}>>\chi_{\infty }$, from Formula (3) we obtain

(4) $$\chi^{\prime }=\frac{\chi_{0}}{1+{\left(\frac{H}{H_{r}}\right)}^{2}}$$

In the field $H=H_{r}$ at α=0 the AC susceptibility takes the value

(5) $$\chi^{\prime }\left(H_{r}\right)=\chi_{\infty }+\frac{\chi_{0}-\chi_{\infty }}{2};\;\chi^{\prime \prime }\left(H_{r}\right)=-\frac{\chi_{0}-\chi_{\infty }}{2}$$

i.e., the real part of the susceptibility, which depends on the magnetic field, decreases by a factor of 2 compared to the value at H=0.

**Figure 8:**
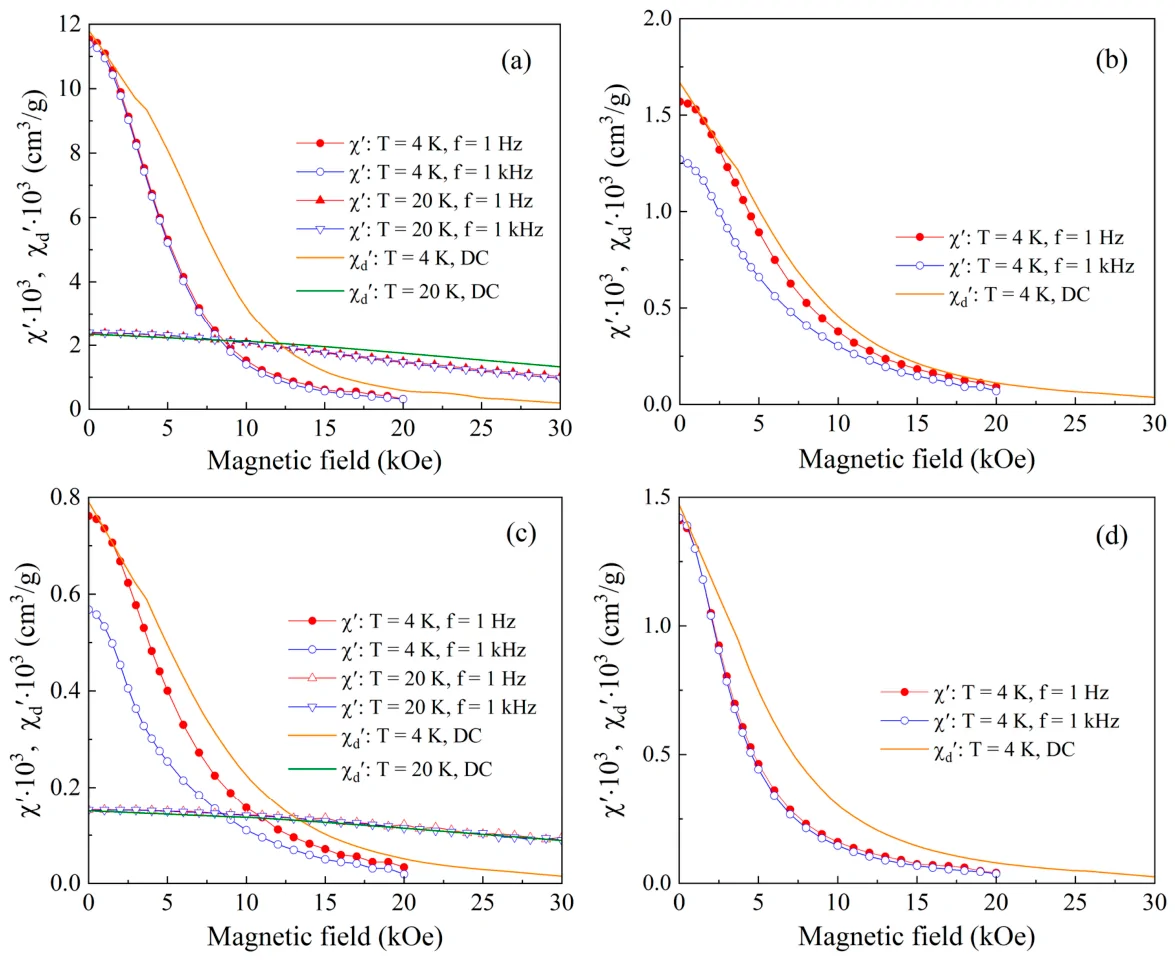
The magnetic field dependences of the real part of the AC susceptibility of doped holmium titanates, measured at frequencies of 1 Hz and 1 kHz at the temperatures of 4 K and 20 K: Ho_2_Ti_2_O_7_—(**a**); Ho_0.25_Y_1.75_Ti_2_O_7_—(**b**); Ho_0.1_Y_1.9_Ti_2_O_7_—(**c**); Ho_0.5_Bi_1.5_Ti_2_O_7_—(**d**).

According to Formula (3), an approximation of the field dependences of the AC susceptibility for samples of titanates Ho_2−*x*_*M_x_*Ti_2_O_7_ was carried out at frequencies f = 1 Hz and 1 kHz. The results are shown in Figure 8. In addition to the dependencies for $\chi^{\prime }$, Figure 8 also shows the field dependencies of the differential susceptibility ${\chi^{\prime }}_{d}=dM\left(H\right)/dH$, which are obtained by differentiating the magnetization curves shown in Figure 4.

Application of a DC magnetic field results in a very significant decrease in $\chi^{\prime }$. The appearance of the $\chi^{\prime }\left(H\right)$ dependences measured at *T* = 4 K and 20 K differs, which is a consequence of the strong dependence of $H_{r}$ on temperature. The frequency dependence of $\chi^{\prime }$ is manifested, in particular, in the fact that the values of $\chi^{\prime }\left(H=0\right)$, measured at different frequencies, differ significantly for titanates doped with yttrium. For the bismuth-doped Ho_0.5_Bi_1.5_Ti_2_O_7_ titanate and undoped holmium titanate, no noticeable frequency dependence was found. For all titanates studied, an approximate equality $\chi^{\prime }\left(H=0\right)\approx {\chi^{\prime }}_{d}\left(H=0\right)$ is observed. This equality indicates that in the studied titanates there are no relaxation times exceeding ~$1/\left(2\pi f\right)$ = 0.16 s. The behavior of the $\chi^{\prime }\left(H\right)$ dependences, measured at a frequency of 1 Hz, and ${\chi^{\prime }}_{d}\left(H\right)$ is similar, and for the Ho_0.25_Y_1.75_Ti_2_O_7_ sample, almost complete coincidence of these dependences is observed. For samples Ho_0.1_Y_1.9_Ti_2_O_7_ and Ho_0.5_Bi_1.5_Ti_2_O_7_, the difference between the dependencies of $\chi^{\prime }\left(H\right)$ and ${\chi^{\prime }}_{d}\left(H\right)$ exceeds the measurement error.

**Figure 9:**
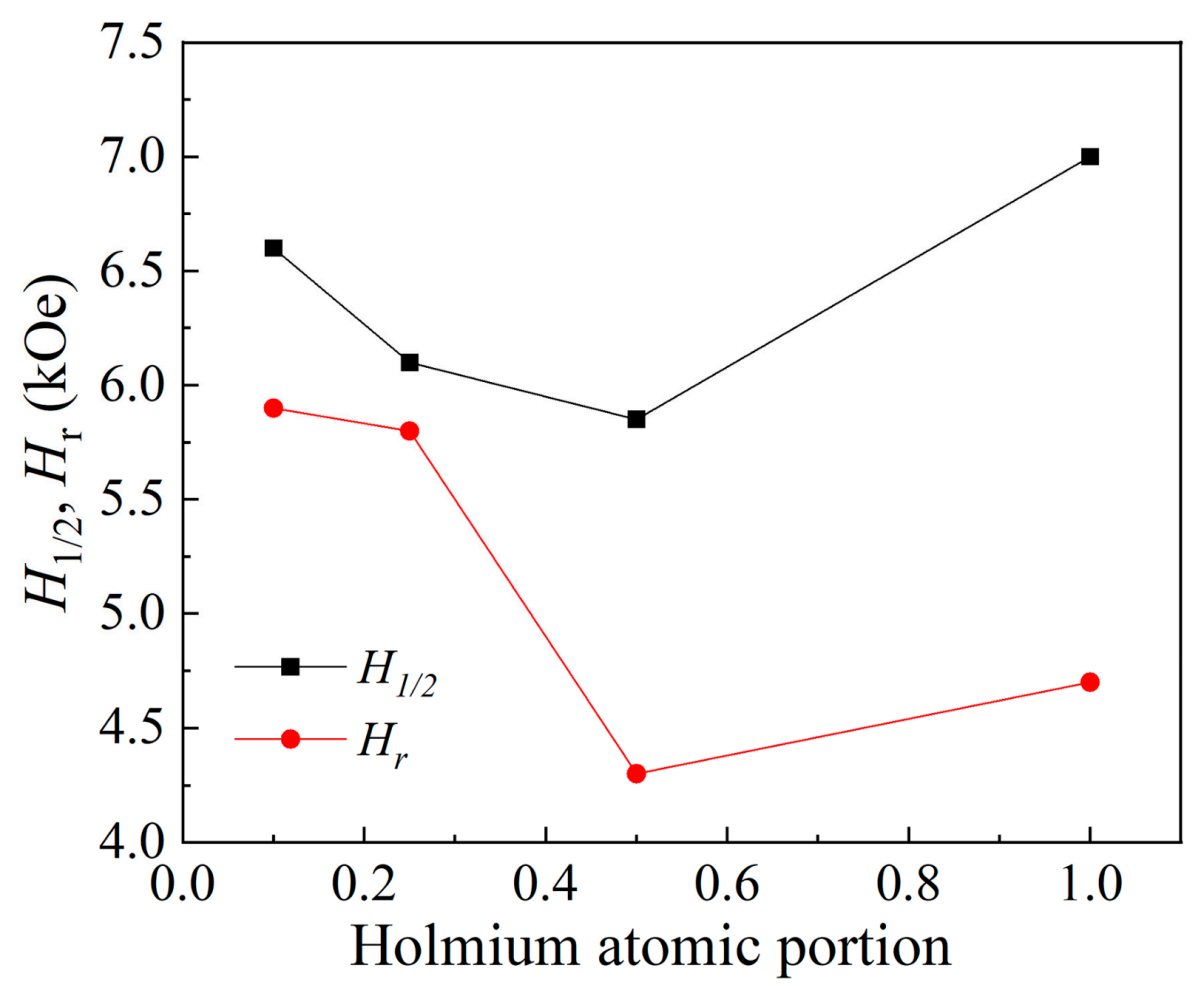
The dependencies of *H*_1/2_ and *H_r_* fields on the holmium atom content for *T* = 4 K. The data for *H_r_* have been obtained at the frequency of *f* = 1 Hz.

It is of interest to find out whether there is a correlation between the fields $H_{r}$ and $H_{1/2}$, where $H_{1/2}$ is the field in which ${\chi^{\prime }}_{d}$ decreases by a factor of 2 compared to the value at H=0, where ${\chi^{\prime }}_{d}\left(H_{1/2}\right)=\frac{1}{2}{\chi^{\prime }}_{d}\left(0\right)$. The comparison result is presented in Figure 9. It can be noted that in a field equal to $H_{r}$, the magnetization *M* is 0.7 of the value *M*(*H* = 30 kOe) for undoped Ho_2_Ti_2_O_7_, from 0.63 to 0.53 for yttrium-doped titanates, and 0.35 for the bismuth-doped titanate Ho_0.5_Bi_1.5_Ti_2_O_7_. Therefore, it can be concluded that the correlation between $H_{r}$ and $H_{1/2}$ is present, but not universal.

## 6. Conclusions

Samples of rare earth titanate pyrochlores Ho_2−*x*_*M_x_*Ti_2_O_7_ (*M*—Bi, Y), with values of *x* equal to 0, 1.5, 1.75, and 1.9, have been synthesized. Measurements of the magnetization curve and the dependence of AC magnetic susceptibility on the DC field strength and on frequency were performed. Temperature dependences of magnetic relaxation times were determined in the range of 1.8–20 K. It is shown that to describe the frequency dependence of AC susceptibility, two relaxation times are required over the entire temperature range. Measurements of the real part of the AC susceptibility as a function of DC magnetic field strength were performed. It is shown that these dependencies satisfy the modified Cole–Cole formula [20]. As the DC magnetic field increases, the AC susceptibility decreases greatly. For several compositions, the field $H_{r}$ is determined in which the real part of the AC susceptibility decreases by two times compared to the value at *H* = 0. It has been established that the $H_{r}$ field increases sharply with increasing temperature. A dependence of *H_r_* that increases rapidly with rising temperature was observed both for the undoped Ho_2_Ti_2_O_7_ titanate and for all doped compositions. The influence of AC field frequencies on this dependence was found to be insignificant. A correlation was established between the $H_{r}$ and the $H_{1/2}$ field, in which the differential DC susceptibility is reduced by half. The field dependences of the real part of the AC susceptibility and the differential DC susceptibility are similar, but do not coincide.

At temperatures above 10 K, the nature of the temperature dependence of the spin relaxation time changes abruptly. The presence of two spin relaxation times and their temperature dependence at temperatures of a few Kelvins are consistent with a model of magnetic monopole motion. At temperatures above 10 K, a single-ion thermally activated relaxation process takes place.

## Figures and Tables

**Table 1:** Crystal lattice parameters and density of compounds.

Compound	*a* (Å)	Distance R(Bi,Y)-R for the Position with the Coordination Number 6 (Å)	Distance R(Bi,Y)-R for the Position with the Coordination Number 12 (Å)	Scherrer Grain Size (Å)	*ρ* (g/cm^3^)
Ho_2_Ti_2_O_7_	10.0940(1)	3.57	6.18	730	6.95
Ho_0.5_Bi_1.5_Ti_2_O_7_	10.2742(1)	3.63	6.29	700	7.38
Ho_0.5_Y_1.5_Ti_2_O_7_	10.0914(2)	3.57	6.18	520	5.47
Ho_0.25_Y_1.75_Ti_2_O_7_	10.0887(2)	3.57	6.18	470	5.23
Ho_0.1_Y_1.9_Ti2O_7_	10.0909(1)	3.57	6.18	860	5.08

**Table 2:** Parameters for approximating the frequency dependences of AC susceptibility for the Ho_0.25_Y_1.75_Ti_2_O_7_ sample.

*T* (K)	$\chi_{1}^{0}$//$\chi_{2}^{0}$ (cm^3^/g)	$\chi_{1}^{\infty }$//$\chi_{2}^{\infty }$ (cm^3^/g)	$\tau_{1}$//$\tau_{2}$ (s)	$\alpha_{1}$//$\alpha_{2}$
1.8	2.02·10^−3^//1.28·10^−3^	5.01·10^−6^//1.37·10^−5^	2.40·10^−5^//1.01·10^−3^	9.57·10^−4^//3.69·10^−1^
2	1.86·10^−3^//1.18·10^−3^	5.01·10^−6^//1.50·10^−5^	2.44·10^−5^//6.89·10^−4^	5.99·10^−4^//3.88·10^−1^
2.5	1.73·10^−3^//7.57·10^−4^	5.02·10^−6^//1.27·10^−5^	3.33·10^−5^//6.17·10^−4^	3.30·10^−3^//3.02·10^−1^
3	1.44·10^−3^//6.48·10^−4^	5.01·10^−6^//1.14·10^−5^	2.44·10^−5^//3.76·10^−4^	6.04·10^−4^//2.81·10^−1^
4	1.13·10^−3^//4.39·10^−4^	5.01·10^−6^//8.97·10^−6^	2.82·10^−5^//2.52·10^−4^	5.78·10^−4^//2.19·10^−1^
5	9.50·10^−4^//3.02·10^−4^	5.16·10^−6^//9.48·10^−6^	2.02·10^−5^//2.35·10^−4^	6.95·10^−5^//1.27·10^−1^
6	9.39·10^−4^//1.03·10^−4^	6.03·10^−6^//8.41·10^−6^	3.13·10^−5^//3.40·10^−4^	9.34·10^−2^//5.07·10^−9^
8	7.21·10^−4^//6.05·10^−5^	4.97·10^−6^//2.82·10^−6^	3.83·10^−5^//3.35·10^−4^	6.26·10^−3^//7.26·10^−3^
10	5.98·10^−4^//2.60·10^−5^	4.98·10^−6^//2.79·10^−6^	3.55·10^−5^//2.38·10^−4^	3.15·10^−6^//1.60·10^−7^
15	4.12·10^−4^//3.72·10^−6^	5.14·10^−6^//2.45·10^−7^	5.03·10^−7^//2.26·10^−4^	2.88·10^−6^//5.76·10^−4^
20	3.05·10^−4^//7.24·10^−6^	1.50·10^−5^//5.27·10^−7^	3.70·10^−7^//1.18·10^−4^	3.01·10^−6^//2.05·10^−4^

## Data Availability

The original contributions presented in this study are included in the article. Further inquiries can be directed to the corresponding author.
